# P-1675. Antibiotic Inertia Occurs Frequently in Patients Admitted from the Emergency Department on Guideline Non-Concordant Therapy

**DOI:** 10.1093/ofid/ofae631.1841

**Published:** 2025-01-29

**Authors:** Joseph Creel, Adrianna Elashker, Michael E DeWitt, James R Beardsley, Christopher Ohl, Mary Banoub, Olivia Randazza, Alex D Taylor, John C Williamson, Charles Hartis, Ryan C McCormick, Vera P Luther

**Affiliations:** Wake Forest University School of Medicine, Winston-Salem, North Carolina; Wake Forest School of Medicine, Winston-Salem, North Carolina; Atrium Wake Forest Baptist Health/ Wake Forest University School of Medicine, Winston-Salem, North Carolina; Wake Forest University School of Medicine, Winston-Salem, North Carolina; Wake Forest School of Medicine, Winston-Salem, North Carolina; Atrium Health Wake Forest Baptist Medical Center, Winston Salem, North Carolina; Atrium Health Wake Forest Baptist, Winston-Salem, North Carolina; Atrium Health Wake Forest Baptist, Winston-Salem, North Carolina; Atrium Health Wake Forest Baptist, Winston-Salem, North Carolina; Atrium Health Wake Forest Baptist, Winston-Salem, North Carolina; Prisma Health, Columbia, South Carolina; Wake Forest University School of Medicine, Winston-Salem, North Carolina

## Abstract

**Background:**

Antibiotic (abx) inertia is when inpatient providers continue inappropriate empiric abx started in the emergency department (ED) rather than changing to an appropriate regimen. Studies using hypothetical case scenarios estimate that this occurs in 39% of cases, but the phenomenon has not been studied using actual prescribing data. The objective of this study is to evaluate abx inertia in real-world clinical practice.

**Figure:**
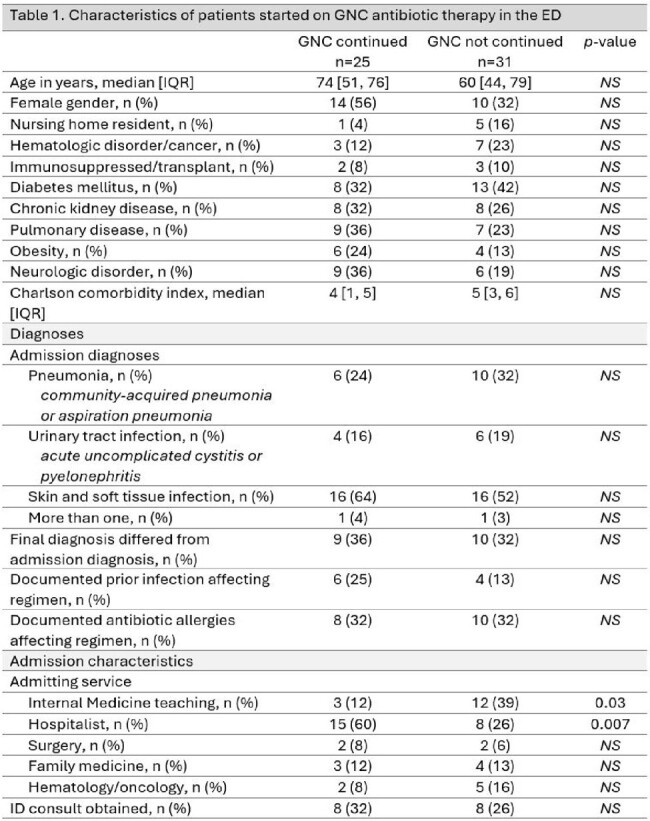

**Methods:**

This retrospective chart review of adult inpatients at an 885-bed tertiary care referral center from January - October 2022 evaluated whether guideline non-concordant (GNC) abx therapy started in the ED was continued upon admission to an inpatient team. Patients admitted with a diagnosis of acute uncomplicated cystitis, pyelonephritis, community-acquired pneumonia (PNA), or skin/soft tissue infection (SSTI) who received abx in the ED were eligible for inclusion. ICU admissions were excluded. Ten patients meeting study criteria were randomly chosen from each month. Abx regimens were compared to those listed in corresponding IDSA guidelines. The primary outcome was the continuation of empiric GNC abx by the inpatient team at the time of admission.

**Figure:**
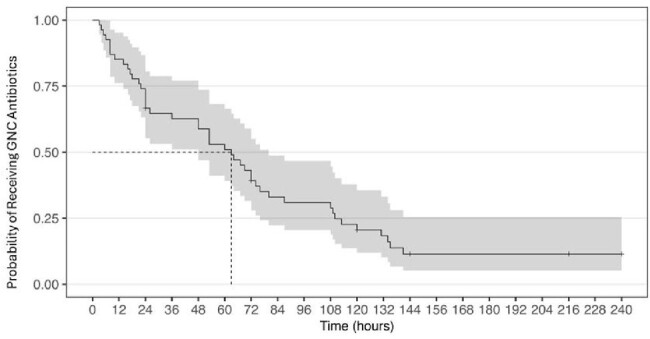

**Results:**

Of the 100 patients evaluated, 56 were started on GNC abx therapy in the ED. Of these, 25 (45%) were continued on GNC regimens. Vancomycin and cefepime, prescribed in 18 (72%) and 10 (40%) cases, were the most commonly continued GNC abx. The majority [23 (92%)] of continued GNC regimens were broader than those listed in the guidelines; 17 (68%) continued an abx not listed in guidelines while also omitting a listed abx. It took a median of 63 hours (95% CI 48-80) for those started on GNC abx in the ED to receive guideline concordant abx (figure 1). There was no difference in age, comorbidities, admission diagnosis, change in diagnosis, prior infections or allergies in patients whose GNC abx were continued versus not. Patients admitted to a hospitalist service were more likely to have GNC abx continued (p < 0.05), table 1.

**Conclusion:**

Abx inertia occurred in almost half of patients started on GNC abx in the ED. Vancomycin and cefepime as well as a specific inpatient service were often associated with continued GNC abx. These observations offer potential targets for antimicrobial stewardship efforts.

**Disclosures:**

**John C. Williamson, PharmD**, Armata Pharmaceuticals: Grant/Research Support|Blue Collar Vaccines and Therapeutics: Board Member|Blue Collar Vaccines and Therapeutics: Ownership Interest|Paratek Pharmaceuticals: Grant/Research Support|ST Pharm Co, Ltd: Grant/Research Support

